# Role of ecological approaches to eliminating schistosomiasis in Eryuan County evaluated by system modelling

**DOI:** 10.1186/s40249-018-0511-7

**Published:** 2018-12-20

**Authors:** Yi Dong, Chun-Hong Du, Yun Zhang, Li-Fang Wang, Jing Song, Ming-Shou Wu, Wen-Can Yang, Shan Lv, Xiao-Nong Zhou

**Affiliations:** 1 Yunnan Institute of Endemic Diseases Control and Prevention, Dali, 671000 Yunnan China; 2 Eryuan Station of Schistosomiasis Control and Prevention, Eryuan, 671200 Yunnan China; 3 National Institute of Parasitic Diseases, Chinese Center for Disease Control and Prevention, Shanghai, 200025 China; 4 Chinese Center for Tropical Diseases Research, Shanghai, 200025 China; 5 WHO Collaborating Centre for Tropical Diseases, Shanghai, 200025 China; 6 National Center for International Research on Tropical Diseases, Ministry of Science and Technology, Shanghai, 200025 China; 7 Key Laboratory of Parasite and Vector Biology, Ministry of Health, Shanghai, 200025 China

**Keywords:** Schistosomiasis, Ecological protection, Transmission block, Elimination, System modelling, Eryuan County, Yunnan Province, China

## Abstract

**Background:**

Schistosomiasis was severely prevalent in Yunnan Province, and it is difficult to achieve its elimination by convention approaches due to complexity of the nature. We explored the comprehensive model to eliminate schistosomiasis in Eryuan County, Yunnan Province, the People’s Republic of China, through integration with the ecological protection programme in Erhai Lake, in order to promote an efficient elimination strategy. We expected that this model is able to be tailored to other local settings, which help achieve the goal of precisely eliminating the disease in Yunnan Province.

**Methods:**

Eryuan County of Yunnan Province was chosen as the study area, where the data on environmental protection activities in Erhai Lake and on the schistosomiasis control programme were collected through different departments of Erhai County government since 2015. System modelling was performed using system dynamics software to establish a simulation model in order to evaluate the effectiveness of intervention activities.

**Results:**

Ecological approaches to control schistosomiasis in Eryuan County consist of three major components: (i) implementing precise interventions to stop schistosomiasis transmission by means of controlling the source of infection, blocking the biological transmission chains and cutting off the route of disease transmission; (ii) employing ecological approaches to improve the co-effectiveness of environmental protection and schistosomiasis prevention in the study area; and (iii) strengthening the professional skills of personnel involving in the schistosomiasis control programme. Simulation results showed that this strategy could speed up the progress of schistosomiasis control programme moving from the control stage to the elimination stage.

**Conclusions:**

Ecological approaches implemented in schistosomiasis endemic areas of the Eryuan region are able to improve the co-effectiveness of environmental protection and schistosomiasis control, providing a new avenue for eliminating schistosomiasis thanks to the application of precise interventions.

**Electronic supplementary material:**

The online version of this article (10.1186/s40249-018-0511-7) contains supplementary material, which is available to authorized users.

## Multilingual abstracts

Please see Additional file 1 for translations of the abstract into the five official working languages of the United Nations.

## Background

The prevalence of schistosomiasis is closely related to factors of the natural environment and socio-economic characteristics [1–4]. Currently, the goal of the national schistosomiasis control programme in China has shifted into transmission elimination in accordance with the 13th Five-Year Plan (2016–2020) [5]. With the rapid economic development in the country, an improved ecological system and environmental protection are essential to achieving sustainable development in rural areas. It is also important to implement precise schistosomiasis control measures in the elimination programme, which need to be tailored to local settings in line with local environmental protection policies [6, 7]. For this reason, all activities in the national schistosomiasis elimination programme must be compatible with China’s ecological construction and environmental improvement strategy. On the basis of rational exploitation which means resources exploitation should be evaluated and planed reasonably, and better utilisation of resources, a new model has been developed, which combines the benefits of environmental protection and schistosomiasis elimination in order to achieve a win-win target for the schistosomiasis control programme with economic, environmental and social benefits [8–11].

Eryuan County, located in the northwest of the Yunnan Province and north to the Erhai Lake, is the upstream water source of Erhai Lake and is an important part of the Erhai Ecological Economic Zone [12]. Eryuan was one of the severe endemic areas for schistosomiasis japonica in Yunnan Province, which reflected in the report of The World Bank Loan project on schistosomiasis in Eryuan County in 2001 that there were 2026 hm^2^ area with *Oncomelania* spp. infested and 29 425 patients in Eryuan County, the infection rate of local people was 16.53%. In order to control schistosomiasis effectively, a comprehensive control strategy with an emphasis on the control of infection sources has been implemented in the endemic area since 2004. As a result, the criteria of schistosomiasis transmission control were achieved in the county in 2015.

In recent years, the pollution load in Erhai Lake has reached its limit, with the population increasing significantly and the total amount of source water decreasing around Erhai Lake. In 2016, the Eryuan County government initiated an intensified ecological programme to protect Erhai Lake by implementing various actions, including those to reduce pollution sources, ecological restoration through water saving and water management, speeding up of sewage interception, and so on. All of these actions have created new opportunities and challenges to implementing the schistosomiasis elimination programme in the local communities.

System dynamics was founded in 1956 by Forrester based on feedback control theory, it is a kind of science used to describe the behaviour of the complex dynamical systems, usually by employing differential equations or difference equations [13], and it has been widely used in the fields of sociology, economy, management, resources and environment, but less in the fields of medicine and public health [14–21]. It is a method for analysing and understanding the dynamic behaviours of complex systems in virtue of computer simulation technology, and has incomparable advantages in the study of complex nonlinear systems.

In this study, we explored an innovative system model for schistosomiasis elimination in Erhai Lake in combination with ecological approaches based on the characteristics of schistosomiasis transmission in Eryuan County. The system modelling method was used to evaluate and predict the effectiveness of interventions, based on the outcomes of the multifarious interventions applied in the study.

## Methods

### Study area

Eryuan County, Dali Bai Autonomous Prefecture, Yunnan Province, the People’s Republic of China was selected as the study area, in order to observe the efficacy of ecological protection approaches integrated with the schistosomiasis elimination programme.

Eryuan is located in the northwest of Yunnan, covering an area of 2614 km^2^: The landscape of the county consists of 88.4% of mountainous and 11.6% of dam areas. The county is characterised by a clear dry-wet season, abundant sunlight, obvious stereoscopic climate and regional microclimate. Eryuan has a typical agricultural landscape so that the government attaches great importance to modern agricultural development. In the first half of 2017, the total income of the county was estimated to be 2.746 billion Chinese yuan (CNY) and the annual net income per capita of peasants was estimated to be 6151 CNY. As the county being located around the upstream water source of Erhai Lake, the local government implements strict actions on ecological environment protection as a local regulation since 2014.

**Fig. 1:**
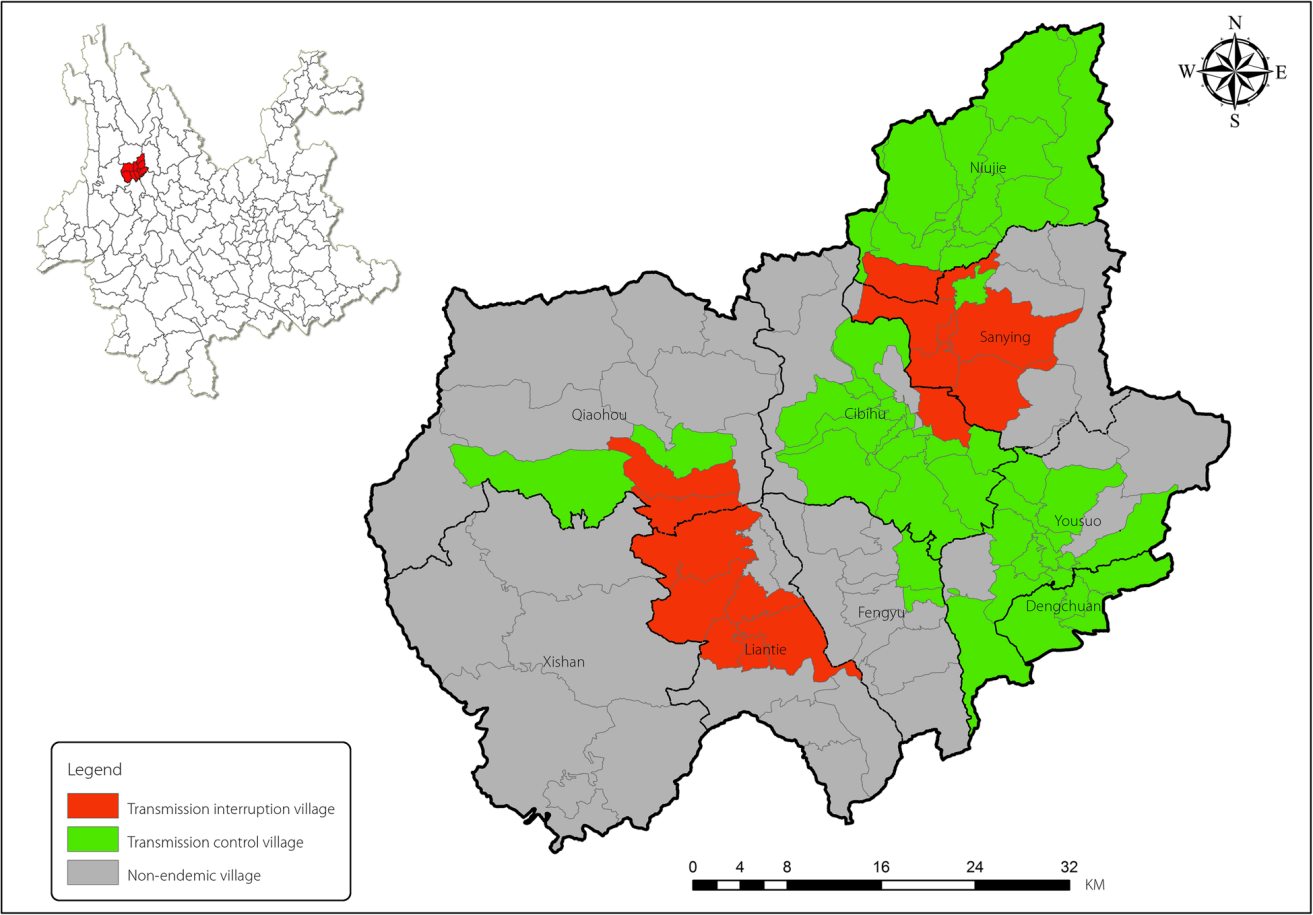
Schematic map of the schistosomiasis epidemic in Eryuan County

Eryuan is a hill-type endemic area for transmission of schistosomiasis japonica with historically severe endemic records. Schistosomiasis was endemic in 382 natural villages and 56 administrative villages of 8 townships (see Table 1). The county has reached the criteria of transmission control in all administrative villages in 2015. A total of 40 administrative villages out of 56 endemic villages achieved the criteria of transmission interruption and the remaining 16 villages achieved transmission control with schistosomiasis prevalence of less than 1%. Since 2015, no case of infected resident or infected cattle has been reported in the entire county. Since 2007, no positive *Oncomelania* spp. snail, as an intermediate snail host of *Schistosoma japonicum*, has been found, although *Oncomelania* spp. snails are still distributed with an infested area of around 2.86 million square meters recorded in 2017 (see Fig. 1).

**Table 1:** Status of schistosomiasis in Eryuan County, 2017

Township name	No. of endemic villages	Population in endemic township	No. of bovine (x1000)	Snail area (hm^2^)	No. of transmission interrupted villages	No. of transmission controlled villages
Dengchuan	4	16 889	4.2	6.13	4	0
Yousuo	12	51 719	10.3	18.33	12	0
Niujie	7	15 948	3.7	12.94	6	1
Sanying	7	34 304	13.8	60.00	1	6
Fengyu	2	5424	1.1	0.48	2	0
Liantie	6	12 040	7.9	136.05	0	6
Qiaohou	4	7660	3.1	20.13	2	2
Cibihu	14	47 176	9.2	31.84	13	1
Total	56	191 160	53.2	285.89	40	16

### Interventions

#### Ecological protection measures

In accordance with the local government taking strict measures to protect the water resources environment around Erhai Lake, seven major approaches of the Erhai conservation programme have been implemented: (i) continual promotion of river basin remediation, (ii) treatment of two kinds of pollution in villages and towns, (iii) reduction of source pollution, (iv) ecological restoration involving water saving and flood control, (v) speed of sewage interception and pollution control projects, (vi) comprehensive law enforcement supervision of the river basin, and (vii) involvement of all people in the Erhai conservation programme, so that the pollution load into the lake is reduced significantly and the ecological environment of Erhai Lake is effectively improved.

#### Schistosomiasis elimination

According to the 13th Five-Year Plan (2016–2020) to control schistosomiasis in Yunnan Province, efforts to eliminate schistosomiasis undertaken by the health and livestock sectors mainly comprise chemical snail control, snail control by environment modification, surveillance of human and livestock infections, improvement of water supply and sanitation, and health education including information, education and communication (IEC). In combination with ecological approaches in Erhai Lake Basin since 2016, schistosomiasis elimination efforts have been implemented with a focus on three approaches: (i) controlling the source of parasitic infection, (ii) blocking the biological transmission chains of *S. japonicum*, and (iii) cutting off the route of disease transmission.

### Evaluation

#### Data collection

Data collections were performed from different departments of the Eryuan County government, including departments of health, agriculture, forestry and water conservancy, to obtain data on the Erhai ecological protection programme, schistosomiasis elimination programme and other related data since 2015. To understand economic data, such as family income, interviews with farmers or residents were arranged and the relevant statistics from the statistics department of the local government were also obtained. In particular, data from the schistosomiasis control programme were collected, including detection and treatment of human and livestock infections, snail survey and control, surveillance of human and livestock infections, and health education.

The human infection survey was performed by screening permanent residents using indirect hemagglutination assay (IHA). If the IHA screening was positive, a faecal examination using the Kato-Katz technique would follow, and finally the infection rate would be calculated based on those results. A snail survey is conducted in the systematic sampling method with a check box (box covers an area of 0.1 m^2^) to seize all snails and an anatomic observation under a microscope to check if positive.

The recommended formula for calculating the human infection rate is as follows [3]:

$$ Infection\ rate\ \left(\%\right)=\frac{No. positive\kern0.17em in\; IHA\; screening}{No. tested\; by\; IHA\; screening}\times \frac{No. positive\kern0.17em in\kern0.17em faecal\kern0.17em exams}{No. tested\; by\; faecal\kern0.17em exams}\times 100\% $$

The formula to calculate the density of snails [22] is:

$$ Density\kern0.17em of\kern0.17em live\kern0.17em snails\;\left( No./ 0.1{m}^2\right)= No. live\kern0.17em snail/ No. survey\; box\;\left( 0.1{m}^2\right) $$

#### Modelling analysis

Based on the elements of both the schistosomiasis control programme in Eryuan and the ecological protection programme in Erhai Lake, essential data, covering the annual data of the control works and investment in the schistosomiasis control programme, as well as the evaluation data, covering the annual infectious indicators used to assess the transmission levels in human, animals and intermediate snail host, collected in Eryuan from 2015 to 2017 were used for the modelling analysis, using the system model method developed by Xu, Xie et al. [23–25]. The simulation using system modelling was performed using system dynamics software (Vensim® PLE. Ventana Systems, Inc., United States), which compared the differences between traditional schistosomiasis control efforts and ecological management. Schistosomiasis indices were simulated for both traditional and ecological approaches. A further significance test on regression between analogue value and practical data was conducted using the Statistical Product and Service Solutions (SPSS 10.0, International Business Machines Corp, New York, United States) software to examine the model stability.

##### Modelling the analysis process

System modelling was performed using the following five steps: (i) confirming the purpose of modelling, which involved applying the dynamic simulation to analyse the relationship between the transmission of schistosomiasis and interventions for schistosomiasis elimination using traditional and ecological modes; (ii) drawing the causal feedback system diagram for both traditional and ecological modes (see Additional file 2); (iii) establishing the system dynamics model for schistosomiasis elimination using both traditional and ecological modes; (iv) performing the preliminary evaluation by comparing the actual number of schistosomiasis infection cases and the predicted transmission index based on model simulation; and (v) conducting the simulation assessment on the effectiveness of the integrated schistosomiasis elimination programme in Eryuan County based on the results of the modelling.

##### Model structure

The basic model structure was designed to analyse the investment and benefit of schistosomiasis elimination measures. Generally, the basic model structure covers following components. (i) State variable: project investment in schistosomiasis control programme is the state variable, including investment on health education, health project, livestock project, ecological management. (ii) Auxiliary variable: state variables were decomposed into auxiliary variables related to control interventions. (iii) Risk factor variable: in combination with data collected from schistosomiasis transmission, the auxiliary variables were converted into the risk factors, including water contact, the people infection, livestock infection and so on, by using Vensim table function of linear interpolation method. (iv) Index of transmission: referring to previous research results on weighting for each risk factor in the evaluation, the risk factors were fit into the index of schistosomiasis transmission, and then the model was formulated. Finally, simulation was undertaken by setting the growth rate of state variables in the model. The details of the modelling setting are described below.

##### State variables

The traditional control mode is to integrate efforts carried out by multiple sectors, including the departments of health, agriculture and education, water conservancy, forestry and other departments, in addition to efforts on comprehensive treatment. After achieving the status of schistosomiasis transmission interruption, projects on health, livestock and health education are sustained by the government. At the same time, multi-sectoral integrated ecological management project is launched in Eryuan Country. Therefore, health, livestock, health education and ecological management were chosen as the state variables for this study, using the project investment in Chinese Yuan (CNY) in 2009 as initial data.

##### Causal chain and auxiliary variables

According to the relevant causality, state variables were decomposed into auxiliary variables of control intervention. The main causal chain and auxiliary variables are shown in Additional files 2 and 3.

##### Incremental and fixed parameters

In order to realise the dynamic simulation of the model, an extension analysis timeline was needed to set the increment of state variables and rate of growth. Referring to Eryuan’s investment from 2009 to 2017, the increment rate was set with its unit as the year (see Additional file 3).

##### Risk factors and index of transmission

Converting relevant variables into risk factors was performed using the table function of Vensim®. The table function is an important feature of system dynamics to establish the nonlinear relationship between two variables, especially soft variables.

Through the table function of Vensim®, the variables were transformed from control interventions to risk factors, such as risk of human infection, risk of livestock infection, contagious water contact and area of snail factor, as well as the input factor on research and development (R&D) on control technology and regulated disposal rate. Then the schistosomiasis transmission index was generated using the above factors according to the combined weight [26, 27] (see Additional file 3).

## Results

### Implementation of combined approaches

Combined approaches of schistosomiasis control and ecological management have been carried out in the Erhai Lake basin since 2016. The activities implemented using the combined approach are summarised below.

#### Controlling the source of infection

The activities to control the source of infection for schistosomiasis transmission comprised forbidding grazing for livestock and poultry along Yong’an River and other major rivers of the county. Areas within 200 m from sides of rivers and around lakeside ponds, such as Cibi Lake; within 500 m of marshland; as well as some specific areas were designated as restricted no-grazing areas. Six large-scale farms were relocated, and another 16 large-scale farms, dairy farms and live pig farms are going to be relocated or permanently shut down. For treatment of livestock and poultry faeces, 12 livestock and poultry faeces collection stations were built, collecting a total of 4 660 tons of faeces monthly.

#### Blocking biological transmission chains

Ecological and environmental modifications of snail breeding areas to permanently block the biological transmission chains of *S. japonicum* were undertaken through the following seven means: (i) transferring of land in the core areas for ecological protection where located along the main river connected to the lake, was performed covering  800 hm^2^ of land on both sides of the river (the transferred land was used for construction of ecological orchards, landscaping, ecological purification ponds, wetlands and other ecological isolation zones according to local conditions); (ii) a water-saving irrigation engineering project was conducted in a land the size of 1333 hm^2^ to improve efficient agricultural production in the Erhai Lake basin; (iii) construction projects to change the snail habitats were conducted, which covers 1000 hm^2^ land, including hardened ditches for 2.5 km and 1.7 km of ploughing road; (iv) ecological management was conducted in 176. 67 hm^2^ steep slope by building an economic forest in which Sichuan pepper, walnut, fruit trees, such as pear, pulm and acid papaya, and other tree species, were planted; (v) forest management and protection was implemented in the Erhai Lake basin covering 96 333 hm^2^ of forest land; (vi) returning farmland into forestry was undertaken in areas of 511hm^2^; and (vii) the new Yong'an River of 12 km, the old Yong'an River of 6.5 km and the Luo Shi River of 3.28 km were managed and cleared.

#### Cutting off the route of disease transmission

Water management and emissions reduction projects were implemented by water price reforming, which provides opportunities for improving water saving mechanisms for agricultural irrigation as well as helping to reduce water contact in risk areas for schistosome infections. A total of 14 000 hm^2^ areas for agricultural efficient water-saving and emissions reduction projects in the Erhai Lake basin were undertaken, including building new water intake facilities such as sluices and reservoirs, and improving irrigation facilities by making dry branch pipes, sprinkler and drip irrigation supplies, etc.

#### Improving the ecological environment

The environment has been rehabilitated since 2016. This comprised the constructions of 120 km of ecological sewage interception ditch where sewage could be naturally purified through the established ecological degradation system and 50 multi-ponds, with 46.7 hm^2^ of wetland, were returned to save more wetland, and restoring 80 hm^2^ of the beach and lakeside zone. All of these further reduced snail habitats and thus risk areas of infection.

### Effectiveness of schistosomiasis control

**Fig. 2:**
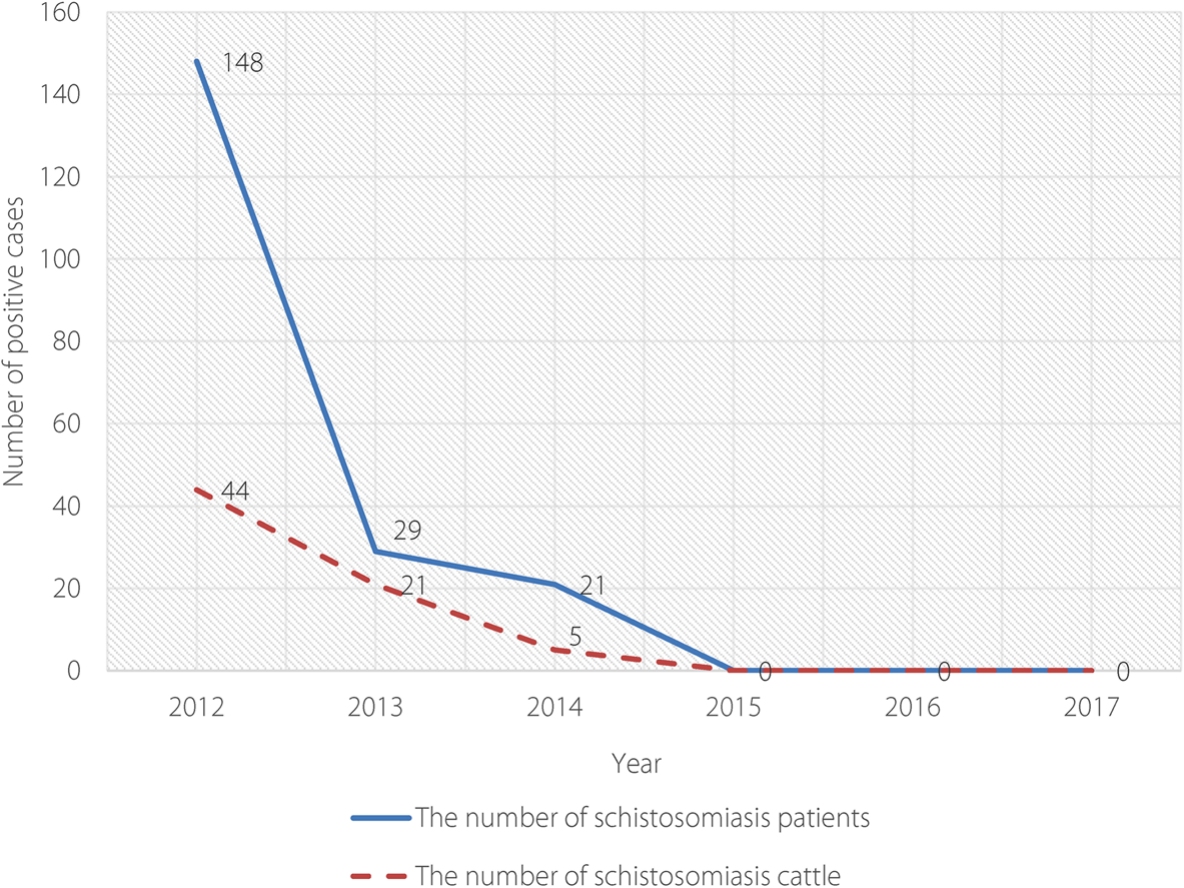
Diagram showing change patterns in the number of schistosomiasis infections both in humans and livestock in Eryuan County, 2012–2017

**Fig. 3:**
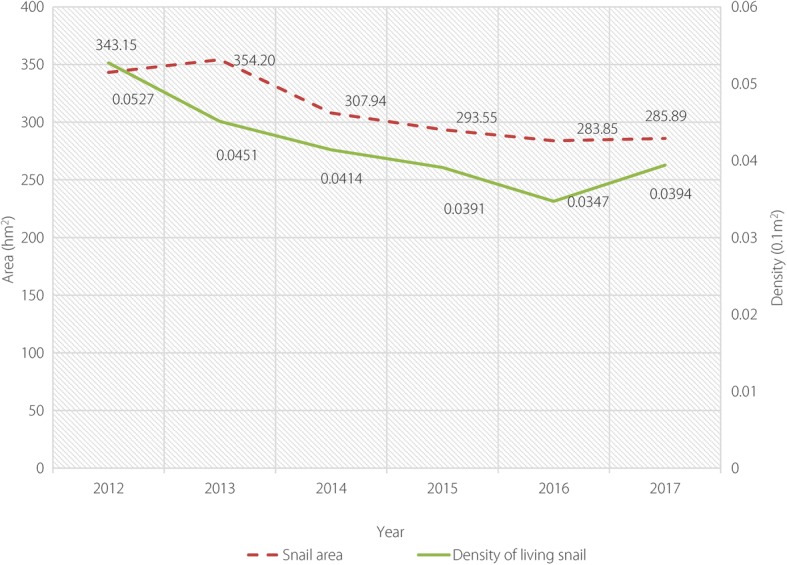
Diagram showing change in the area of *Oncomelania* snail habitats in Eryuan County, 2012–2017

The number of schistosomiasis infections both in humans and cattle was reduced to zero after ecological management approaches were initiated in Eryuan County (see Fig. 2). The area of snail habitats was also reduced by 16.90% compared to that before the ecological management approaches were implemented (see Fig. 3 and Table 2).

**Table 2:** Change patterns in the number of schistosomiasis infections in both humans and livestock in Eryuan County, 2012–2017

Year	Humans					Bovine			Snail infested area (hm^2^)	Density of living snails (per 0.1 m^2^)
	No. tested by immunological exams	No. positive in immunological exams	No. tested by faeces exams	No. positive in faeces exams	Infection rate (%)	No. examined	No. positive	Positive rate (%)		
2012	83 010	13 266	17 339	148	0.14	50 529	44	0.09	343.15	0.053
2013	76 349	6453	15 785	29	0.02	31 411	21	0.07	354.20	0.045
2014	46 256	3969	12 262	21	0.01	16 966	5	0.03	307.94	0.041
2015	55 110	3026	11 211	0	0.00	7208	0	0.00	293.55	0.039
2016	41 350	2171	8983	0	0.00	29 289	0	0.00	283.85	0.035
2017	58 116	3955	9829	0	0.00	30 156	0	0.00	285.89	0.039

### System model for schistosomiasis elimination

Based on the natural characteristics of schistosomiasis transmission, the features of economic and social development in Eryuan County, and the ecological protection practices around Erhai Lake, a combined model of ecological protection and schistosomiasis control in Eryuan County was established using the system model developed using Vensim® PLE software. Two scenarios of control effectiveness were simulated, including a traditional model of schistosomiasis control and an ecological approach model.

In the ecological approach model, the state variables employed included the anti-infection intervention by the health sector, livestock schistosomiasis control by the agriculture sector, health education by the education sector and ecological management by multiple sectors. The variables of acceleration rate were also employed, including annual growth rate and increment of state variables aforementioned. The schistosomiasis transmission index was expressed by incremental variable equation of infested water (e.g. human, animal and snail infections), water contact factor, case surveillance response rate, control technology development index.The auxiliary variables included several aspects of ecological management and various measures of the traditional schistosomiasis control programme. The ecological approach model in Eryuan has been described as schistosomiasis control integrated with ecological management approaches (see Table 3).

**Table 3:** Ecological management measures and their effects on schistosomiasis control

Classification	Ecological management measures	Effect on schistosomiasis control
Control the source of infection	Set no pastoral area	Such measures effectively reduce *Schistosoma* eggs that pollute the environment and prevent the infection of snail from schistosome miracidium.
	Close or transfer cultivation factory	
	Dispose of human and animal excrement	
	Build sanitation toilets	
	Build a large factory to dispose of livestock excrement	
Block the biological transmission chains	Using right circulation of land, change its purpose of use	Such measures can effectively change the suitable habitats of the snail intermediate host, and can effectively reduce or even get rid of snail habitats.
	Water saving irrigation	
	Harden the ditches and roads	
	Develop economic forest	
	Return the plough into forestry, forest land conservation	
	Channel management	
Cut off transmission of the disease	Control the price of water	Such measures change people’s habits, thereby reducing contact oppertunities with contaminated water and thus reducing the risk of infection.
	Collect polluted water	
	Centralise water supply	
	Implement efficient water-saving projects	
Improve ecological environment	Improve the environmental restoration, by building ecological furrow	Such measures reduce the risk areas by declining the infection of humans and livestock, but must pay attention to the diffusion of snails.
	Improve wetlands restoration	
	Reconstruct lakeside area	

**Fig. 4:**
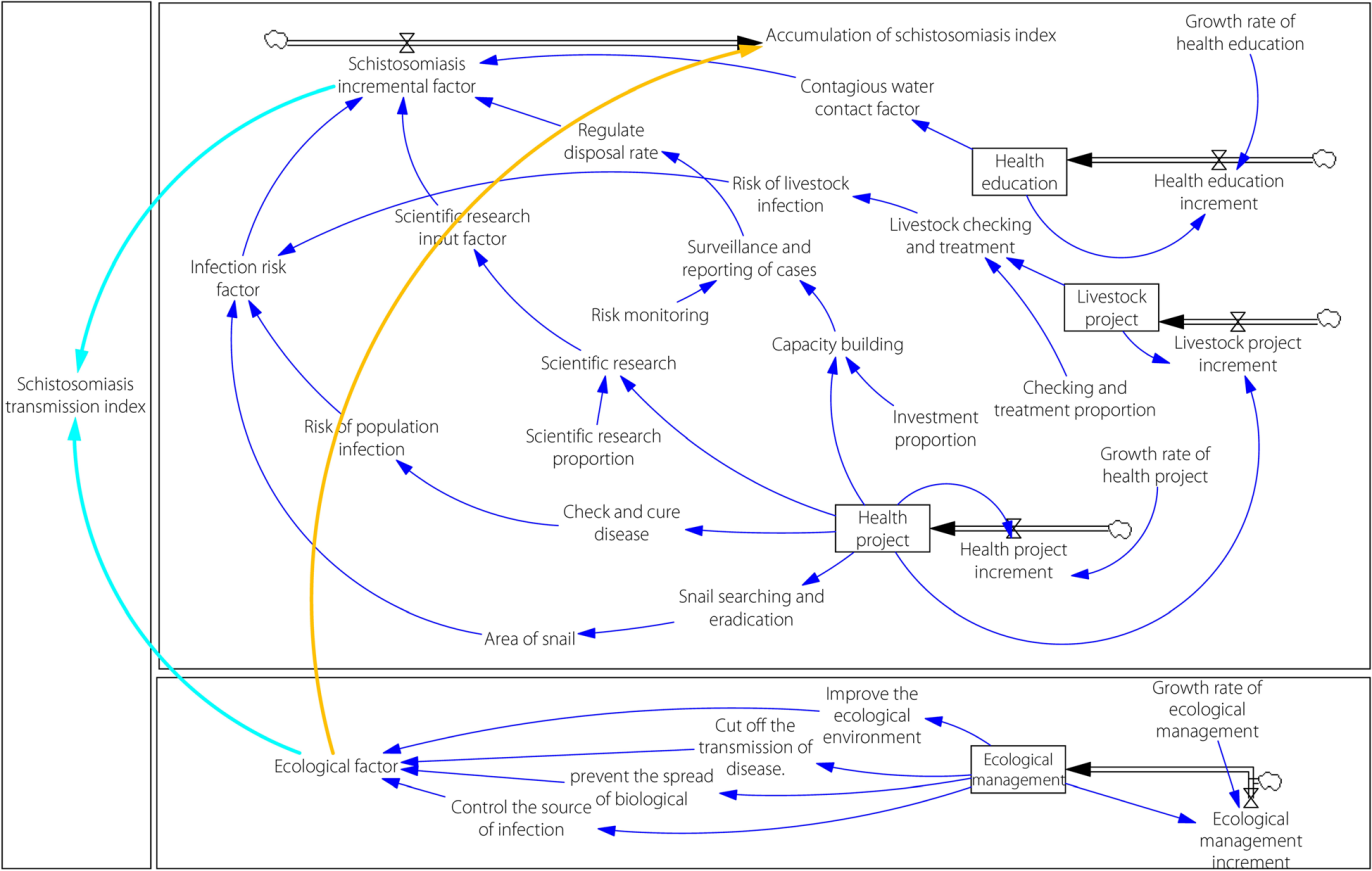
Flow chart showing system modelling using ecological approaches in the schistosomiasis elimination programme in Eryuan County

Based on the system flow chart in the established system dynamics model (see Fig. 4), the ecological approach model of schistosomiasis control in Eryuan County consists of three major components. First, precise interventions to stop schistosomiasis transmission were implemented, which included controlling the source of infection both in humans and livestock, blocking the biological transmission chain and cutting off the route of disease transmission. Second, ecological approaches were undertaken to improve the co-effectiveness of environmental protection and schistosomiasis prevention in the lake area. Third, professional skills of personnel involved in the schistosomiasis control programme were strengthened. The simulation results using the system model showed that the ecological approach model can speed up the progress of schistosomiasis control and its foray into the elimination phase.

**Fig. 5:**
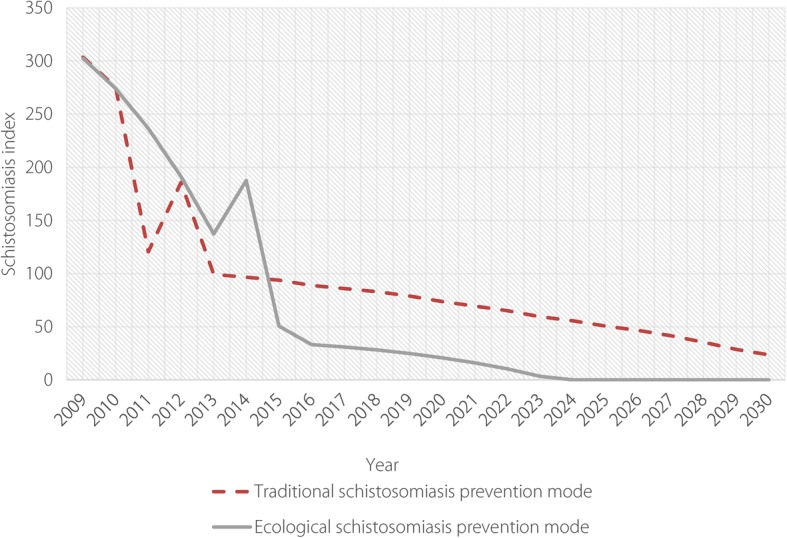
Simulation of schistosomiasis transmission index predicted by system modelling in Eryuan County. The red line shows the change pattern of schistosomiasis transmission index predicted by the traditional schistosomiasis intervention model (model 1), and the grey line shows the change pattern of schistosomiasis transmission index predicted by the ecological approach model (model 2)

By adjusting the combination of relevant variables, we established a simulation calculation for both traditional and ecological models. The results from the system modelling graph showed that the combination model (model 1) integrated with traditional anti-infection interventions, control of livestock schistosomiasis and health education, without ecological inputs and only a small increase in investment, resulted in schistosomiasis declining slowly in terms of the level of schistosomiasis transmission index, thus making it difficult to achieve the elimination target by 2025. However, under the combined model (model 2), the schistosomiasis transmission index can be reduced to a very low level and it is then feasible to achieve schistosomiasis elimination by 2025, especially after effective control of snail areas using ecological approaches and increasing investment in ecological management approaches (see Fig. 5).

### Validation of the dynamic model

In the study, system dynamics model does not make use of evaluation on transmission kinetic parameters and cannot tell the specific numerical value, but according to the weights of correlative factors, it produced schistosomiasis risk index. The comprehensive weight of human and animal infection was the highest, indicating that the predicted risk index is basically equivalent to the past evaluation of index for schistosomiasis elimination [26], so that we compared the similarity between schistosomiasis risk index from the modelling results and the actual number of infections in human and livestock, then verify whether the changes in schistosomiasis transmission risk are consistent with the real situation.

**Fig. 6:**
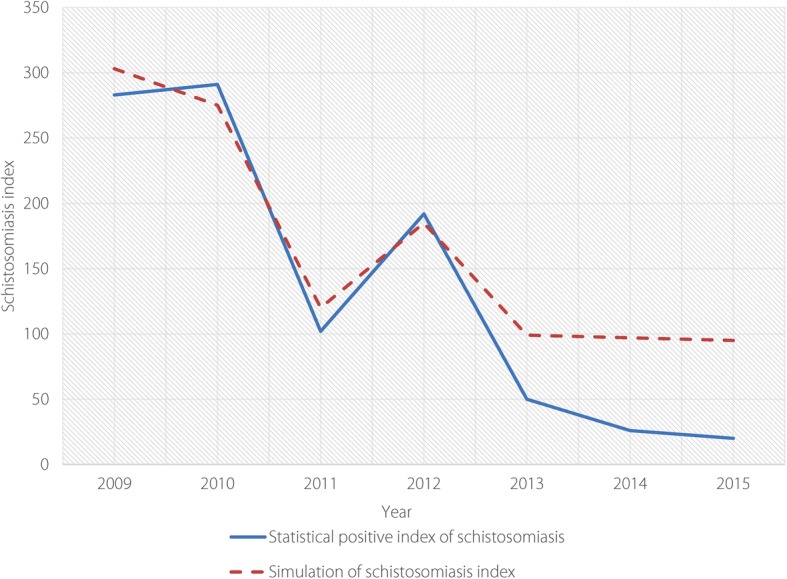
Verification result of simulation using system modelling in Eryuan County

The validation results of the dynamic model, which were obtained by comparing the data between the actual reported number of schistosomiasis cases and predicted schistosomiasis transmission index based on the model simulation from 2009 to 2015 in Eryuan County (see Table 4), shown that the curve fitting is of consistency (see Fig. 6), indicating the coherence between curve for the index of model and the real trend of schistosomiasis transmission risk is well fitted. A further significance test showed that the *P*-value was 0.024 in the regression test, which is less than 0.05 (see Additional file 4), indicating that the model was well established. Therefore, the results of well-fitted linear regression between analogue value and practical data indicated that the established linear relation is basically anastomotic and its tendency is unanimous, so the model can use for further analysis (see Table 4 and Fig. 6).

**Table 4:** Schistosome infections and their simulated values in Eryuan County, 2009–2015

Year	No. of human schistosome infections	No. of bovine schistosome infections	Total schistosome infections	Simulated value
2009	122	161	283	303
2010	193	98	291	275
2011	64	38	102	120
2012	148	44	192	185
2013	29	21	50	99
2014	21	5	26	97
2015	0	0	0	95

## Discussion

To expand our ability to predict and mitigate infectious diseases, including schistosomiasis, it is proposed that a comprehensive method is used to analyse disease dynamics associated with multi-impact factors from different dimensions by understanding the entirety of a system’s components and the complexity of the components’ interrelated behaviours [7]. Particularly, the transmission of schistosomiasis, a zoonotic parasitic disease, is a dynamic process determined by multiple factors originating from disease pathogens or parasites, snail intermediate host and human populations.

The national schistosomiasis control and elimination programme in China has been designed by the government, with its strategy to integrate with multiple sectors at different levels. Therefore, a new strategy of comprehensive schistosomiasis control dominated by the control of infection sources was put into effect in 2004 [28]. By the end of 2015, the target of transmission control was achieved on schedule [29, 30]. In November 2014, the State Council held a national schistosomiasis control conference, which set the goal of schistosomiasis elimination by 2025 [31]. However, the life cycle of schistosomiasis is very complicated, and the factors influencing the transmission and spread of schistosomiasis are related to many different factors, such as social economy, natural ecology, and so on [32–35]. Therefore, continuous control efforts until schistosomiasis is eliminated are more difficult to achieve at the implementing level [36, 37].

It has been suggested that the most effective way to eliminate schistosomiasis is to adopt a comprehensive control strategy that focuses on controlling sources of infection and adapts to local settings. This adaptation approach is designed on the basis of the following three settings: (i) a reasonable overall implementation of the schistosomiasis elimination programme in line with local conditions; (ii) effectively eliminating the impacts from social and natural factors, e.g. population movement, animal trades, floods, etc., that affect schistosomiasis transmission directly through comprehensively controlling the ecological system in endemic areas; (iii) a sound guidance for people to change their unhealthy lifestyles in order to accelerate the transition of the national schistosomiasis programme from transmission control to transmission elimination, and ultimately achieve the target of schistosomiasis elimination in China [38–42].

Two major outputs were obtained through the study. First, that established system-based dynamic modelling is well fitted to the needs of the investigation. The study showed that both curves of real data and predicted data well matched and the *P*-value in the significance test was less than 0.05, indicating that the simulation value well matched with the actual data. Hence, the established dynamic model can be used to evaluate the simulation efficacy of various modes in the schistosomiasis elimination programme. Second, it will not be feasible to achieve the target of eliminating schistosomiasis by 2025 in Eryuan County if only the traditional approach is used, since the established simulation calculation of both traditional and ecological models indicated that using the traditional model (model 1) without ecological inputs will make it difficult to achieve the elimination target by 2025. However, the combination model integrated with traditional interventions and ecological approaches is feasible to meet the schistosomiasis elimination target by 2025, especially after controlling snail-infested areas effectively using ecological approaches.

At present, China has proposed to set upon a new pathway in terms of sustainable development and green modernisation to establish an ecological system with the concept of respecting, complying with and protecting nature, to build an ecological civilisation and strengthen environmental protection with a view to achieve harmonious and sustainable development between humans and nature. To this end, the schistosomiasis control programme in China has also made efforts to tailor itself to local conditions in order to find a breakthrough that is compatible with China’s policy of ecological construction and environmental improvement [43, 44]. Therefore, it is feasible to integrate schistosomiasis interventions with ecological management approaches, which develop new opportunities to improve the schistosomiasis elimination strategy in terms of teamwork capability, technology precision and target identification, in line with local economic and social development goals [45–47].

Although it is possible for Eryuan County to eliminate schistosomiasis by 2025, as long as the ecological approach can be added to the programme, it is essential to consider the following potential risk factors. First, Eryuan County is an underdeveloped area dominated by an agricultural economy, in which the proportion of agricultural population accounting for 92% of the total population is large, and the industrial structure is relatively simple, meaning that the resources put into the schistosomiasis elimination programme are limited. Second, there are many ditches of rivers, lakes and ponds in Eryuan County and the rice paddies found in most areas are suitable for snail breeding due to the county’s warm climatic conditions throughout the entire year, which can reintroduce the schistosomiasis epidemic easily. Third, despite the current low-level endemicity of schistosomiasis, local residents still face a huge threat of schistosomiasis infection due to the routine surveillance system not being sensitive enough, integrated management not being highly integrated, and the efficiency of special funds input not being high enough. Forth, contradictions exist between economic development and ecological environment degradation, and between ecological protection and environmental pollution caused by large-scale chemical mollusciciding, so not all areas easily lend themselves to interventions integrated with ecological approaches [48].

In spite of system dynamics being remarkably effective when applied in many different fields [49], it still has some room for improvement. For instance, parameter estimation is not strength enough in the established system dynamics, however, by integrating existing models, such as the economic model, structural equation, mathematical model, engineering mathematical model and the parameter estimation and function simulation, etc., it is still possible to find the key influence structure and the rule of evolution in the disease transmission. In this study, the ecological mode in schistosomiasis elimination established in Eryuan County through the system dynamics model is able to simulate and evaluate the long-term effect, which means it is possible to predict the changing trends of schistosomiasis transmission risk in different modes, rather than by accurately predicting the future of precise numerical. It is noted that there are many exogenous variables in the model, the function relationship between the variables is estimated and it is not very accurate based on the predicted results, e.g. infection rate and number of cases, but the overall trend prediction meets the purpose of the study. It is hope that we are able to integrate the key parameters of schistosomiasis transmission model [50–53] with the system dynamics model to improve the accuracy of the prediction and evaluation of schistosomiasis transmission.

## Conclusions

Eryuan County, as an important part of the Erhai Ecological Economic Zone, has the goal of achieving schistosomiasis transmission interruption by 2020 and elimination by 2025 as determined in the 13th Five-Year Plan for the national schistosomiasis elimination programme. System modelling demonstrated that it is possible for Eryuan County to eliminate schistosomiasis by 2025, as long as the ecological approach is added to the programme. However, in the implementing level, it is essential to consider potential risk factors that may affect the achievement process of the expected goal. These include: the limited resources put into the disease elimination programme, the existing environment being suitable for snail breeding, and the conflict between economic development and ecological environment protection.

This study also demonstrated that system modelling is able to predict the transmission patterns of schistosomiasis along with interventions at the county level, and to provide assistance in designing the mitigation strategy for the schistosomiasis elimination programme.

## Additional files

Additional file 1: Multilingual abstracts in the five official working languages of the United Nations. (PDF 665 kb)

Additional file 2: Diagram of main causal chains. (DOCX 43 kb)

Additional file 3: List of equations for statistic and function analyses in model development. (DOCX 24 kb)

Additional file 4: Statistically significant results obtained with SPSS. (DOCX 21 kb)

## Abbreviations

CNY: Chinese yuan
IHA: Indirect hemagglutination assay IEC information, education and communication
R&D: Research and development
