# Supplementary material for: Role of ecological approaches to eliminating schistosomiasis in Eryuan County evaluated by system modelling
Source: Infect Dis Poverty. 2018 Dec 20;7:129. doi: 10.1186/s40249-018-0511-7 (PMC6309097; doi:10.1186/s40249-018-0511-7)
Supplement: Supplementary file 1 — Multilingual abstracts in the five official working languages of the United Nations. (PDF 665 kb) [file 40249_2018_511_MOESM1_ESM.pdf]

Translation of the abstract into the five official working languages of the United Nations

دور النهج الإيكولوجية للقضاء على داء البلهارسيات في مقاطعة إريوان التي تم تقييمها بواسطة نمذجة النظام

يي دونج ، وتشون-هونغ دو ، يون تشانج ، لي فانغ وانغ ، جينغ سونغ ، مينغ-شو وو ، ون كان يانغ ، شان لو ، شياو نونغ تشو

الملخص
خلفية: تنتشر البلهارسيا انتشارا شديدا في مقاطعة يوننان، ومن الصعب القضاء عليها بنهج الاتفاقية نظراً للطابع المعقد للطبيعة. لقد استكشفنا النموذج الشامل للقضاء على داء البلهارسيات في محافظة إريوان ، مقاطعة يونان ، جمهورية الصين الشعبية ، من خلال الاندماج مع برنامج الحماية البيئية في بحيرة Erhai ، من أجل تعزيز استراتيجية القضاء الفعال. كنا نتوقع أن يكون هذا النموذج قابلا للتكيف مع الظروف المحلية الأخرى ، والتي تساعد على تحقيق هدف القضاء على المرض على وجه التحديد في مقاطعة يوننان.
الأساليب: "مقاطعة اريوان في محافظة يوننان" اختيرت كمنطقة الدراسة، حيث تم جمع البيانات المتعلقة بأنشطة حماية البيئة في بحيرة أراى وبرنامج مكافحة مرض البلهارسيا من خلال الإدارات المختلفة من حكومة مقاطعة أرايمنذ عام 2015. أجري نظام نمذجة باستخدام البرمجيات ديناميات النظام إلى إنشاء نموذج محاكاة لتقييم فعالية أنشطة التدخل.
نتائج: النهج الإيكولوجي لمكافحة البلهارسيا في مقاطعة اريوان تتألف من ثلاثة مكونات رئيسية: (ط) تنفيذ تدخلات محددة لوقف انتقال البلهارسيا عن طريق التحكم في مصدر العدوى، حجب سلاسل انتقال البيولوجية وقطع الطريق لانتقال المرض؛ (ثانيا) تستخدم النهج الإيكولوجي لتحسين كويفيكتيفينيس لحماية البيئة والوقاية من مرض البلهارسيا في منطقة الدراسة؛ و (ثالثا) تعزيز المهارات المهنية للأفراد المتورطين في برنامج مكافحة البلهارسيا. وأظهرت نتائج المحاكاة أن هذه الاستراتيجية يمكن أن تسرع من تقدم برنامج مكافحة داء البلهارسيات من مرحلة المراقبة إلى مرحلة الإزالة.
الاستنتاجات: النهج الإيكولوجية المنفذة في مناطق موبوءة بداء البلهارسيا من منطقة اريوان قادرين على تحسين كويفيكتيفينيس لمراقبة البيئة حماية والبلهارسيا، إيجاد وسيلة جديدة للقضاء على داء البلهارسيا وبفضل تطبيق التدخلات دقيقة.

Translated from English version into Arabic by Kowthar Alasady, proofread by Nader Hassan
, through

TRANSLATORS
WITHOUT BORDERS

系统动力学建模评价洱源县消除血吸虫病过程中生态措施的作用

董毅，杜春红，张云，王丽芳，宋静，吴明寿，杨文灿，吕山，周晓农

摘要
引言：云南省属于血吸虫病流行区，由于自然条件复杂，很难用常规方法消除血吸虫病危害。通过结合洱海生态保护规划，探索云南省洱源县消除血吸虫病的综合防治模式，以促进有效防治对策的制定。我们期望该模式能适合当地其他区域，有助于云南省精确消除血吸虫病目标的实现。
方法：选取云南省洱源县为研究区域，收集 2015 年以来的血吸虫病防治资料和洱海区域实施的生态保护措施的数据。采用系统动力学软件进行系统建模，建立仿真模型来评价干预活动的有效性。
结果：洱源县血吸虫病的生态防治主要包括三个组成部分：一是通过控制传染源、阻断生物传播链、切断疾病传播途径，实施精准干预以阻止血吸虫病传播；二是采用生态干预措施提高研究地区环境保护和血吸虫病防治的协同效应；三是加强血防专业人员的专业技能。仿真结果表明，该策略可以加快血吸虫病从控制到消灭阶段的进程。

结论：洱源县血吸虫病流行区实施生态干预措施，提高了环境保护和血吸虫病防治的协同效果，为血吸虫病的防治提供了新途径。

Translated from English version into Chinese by Yi Dong

Rôle des approches écologiques dans l'élimination de la schistosomiase dans le comté d'Eryuan : évaluation par modélisation systémique.

Yi Dong, Chun-Hong Du, Yun Zhang, Li-Fang Wang, Jing Song, Ming-Shou Wu, Wen-Can Yang, Shan Lv, Xiao-Nong Zhou

Résumé

Contexte: La prévalence de la schistosomiase est très élevée dans la province du Yunnan, et parvenir à son élimination par des approches conventionnelles se révèle difficile en raison de la nature complexe de cette maladie. Nous avons exploré le modèle complet d'élimination de la schistosomiase dans le comté d'Eryuan, dans la province du Yunnan, en République populaire de Chine, à partir du programme de protection écologique du Lac Erhai, afin d'arriver à une stratégie d'élimination efficace. Nous attendions de ce modèle qu'il puisse être adapté à d'autres endroits du Yunnan, dans le but précis de contribuer à l'élimination de la maladie dans cette province.
Methodes: Nous avons choisi comme zone d'étude le comté d'Eryuan, dans la province du Yunnan, où les activités de protection de l'environnement et le programme de lutte contre la schistosomiase font l'objet d'un recueil de données par différents services des autorités du comté d'Erhai depuis 2015. Une modélisation systémique a été réalisée au moyen d'un logiciel de la dynamique des systèmes pour définir un modèle de simulation visant à évaluer l'efficacité des activités d'intervention.
Résultats: Les approches écologiques utilisées pour lutter contre la schistosomiase dans le comté de Eryuan sont composées de trois éléments principaux: (i) la mise en place d'interventions spécifiques visant à stopper la transmission de la schistosomiase grâce au contrôle de l'origine de l'infection, au blocage des chaînes de transmission biologique et à l'interruption de la voie de transmission de la maladie; (ii) le recours à des approches écologiques pour améliorer l'efficacité conjuguée de la protection environnementale et de la prévention de la schistosomiase dans la zone étudiée; (iii) le renforcement des compétences professionnelles du personnel participant au programme de lutte contre la schistosomiase. Les résultats de la simulation ont montré que cette stratégie pourrait permettre au programme de lutte contre la schistosomiase de progresser plus rapidement, passant du stade de lutte au stade d'élimination.
Conclusions: Les approches écologiques mises en œuvre dans les zones d'Eryuan où la schistosomiase est endémique peuvent améliorer l'efficacité conjuguée de la protection environnementale et de la lutte contre la schistosomiase, et offrir un nouveau moyen pour éliminer la schistosomiase grâce à l'application d'interventions spécifiques.

Translated from English version into French by Cendrine Strevens, proofread by Suzanne Assenat, through

TRANSLATORS
WITHOUT BORDERS

Роль экологических подходов к ликвидации шистосомоза в уезде Эрюань на основе системного моделирования

И Дун (Yi Dong), Чунь-Хун Ду (Chun-Hong Du), Юнь Чжан (Yun Zhang), Ли-Фан Ван (Li-Fang Wang), Цзин Сун (Jing Song), Мин-Шоу У (Ming-Shou Wu), Вэнь-Цань Ян (Wen-Can Yang), Шань Лв (Shan Lv), Сяо-Нун Чжоу (Xiao-Nong Zhou)

Аннотация

Справочная информация: Шистосомоз серьёзно преобладал в провинции Юньнань, и сложность его специфики существенно затрудняет ликвидацию заболевания с помощью традиционных подходов. В целях содействия эффективной стратегии ликвидации мы изучили комплексную модель ликвидации шистосомоза в уезде Эрюань, провинция Юньнань, Китайской Народной Республики путём интеграции с программой экологической защиты на озере Эрхай. Мы ожидали, что указанную модель можно будет адаптировать для других местных параметров, что поможет нам достичь цели по ликвидации этого заболевания в провинции Юньнань.

Методы: В качестве области исследования был выбран уезд Эрюань провинции Юньнань, где данные о деятельности по охране окружающей среды на озере Эрхай и о программе контроля шистосомоза были собраны через различные департаменты правительства уезда Эрхай с 2015 года. Системное моделирование выполнялось с использованием программного обеспечения по системной динамике с целью создания имитационной модели по оценке эффективности вмешательства.

Результаты: Экологические подходы для контроля шистосомоза в уезде Эрюань состоят из трёх основных компонентов: (i) осуществление точного вмешательства с целью прекращения передачи шистосомоза посредством контроля источника инфекции, блокирование биологических цепей передачи, а также отрезание маршрута передачи болезни; (ii) использование экологических подходов для совершенствования общей эффективности охраны окружающей среды и предотвращения шистосомоза в районе исследований; кроме того, (iii) укрепление профессиональных навыков персонала с участием в программе контроля шистосомоза. Результаты моделирования показали, что данная стратегия способна ускорить переход программы по контролю шистосомоза из стадии контроля на стадию ликвидации.

Выводы: Экологические подходы, внедрённые в эндемичных районах по шистосомозу в уезде Эрюань, способны улучшить общую эффективность охраны окружающей среды и контроля шистосомоза, предоставив новые возможности для ликвидации шистосомоза благодаря применению точных вмешательств.

Translated from English version into Russian by Liudmila Tomanek, proofread by Olga Madiar, through

TRANSLATORS
WITHOUT BORDERS

El papel de los enfoques ecológicos para eliminar la esquistosomiasis en el condado de Eryuan, evaluado mediante la simulación de sistemas

Yi Dong, Chun-Hong Du, Yun Zhang, Li-Fang Wang, Jing Song, Ming-Shou Wu, Wen-Can Yang, Shan Lv, Xiao-Nong

Resumen

Introducción: La esquistosomiasis se extendió en gran medida en la provincia de Yunnan y es difícil lograr su eliminación mediante los enfoques convencionales debido a la complejidad de la naturaleza. Se ha explorado el modelo integral para eliminar la esquistosomiasis en el condado de Eryuan, provincia de Yunnan, República Popular China, mediante la integración con el programa de protección ecológica en el lago Erhai, con el fin de promover una estrategia de eliminación eficaz. Se espera que este modelo pueda adaptarse a otros entornos locales, lo que ayuda a lograr el objetivo de eliminar con precisión la enfermedad en la provincia de Yunnan.

Métodos: Se eligió el condado de Eryuan de la provincia de Yunnan como el área de estudio, donde los datos sobre las actividades de protección ambiental en el lago Erhai y el programa de control de la esquistosomiasis se recopilaron a través de diferentes departamentos del gobierno del condado de Erhai desde 2015. Se realizó la simulación de sistemas utilizando el software de dinámica de sistemas para establecer un modelo de simulación y evaluar la efectividad de las actividades de intervención.

Resultados: Los enfoques ecológicos para controlar la esquistosomiasis en el condado de Eryuan constan de tres componentes principales: (i) implementar intervenciones precisas para detener la transmisión de la esquistosomiasis mediante el control de la fuente de infección, el bloqueo de las cadenas de transmisión biológica y el corte de la ruta de transmisión de la enfermedad; (ii) emplear enfoques ecológicos para mejorar la eficacia conjunta de la protección ambiental y la prevención de la esquistosomiasis en el área de estudio; y (iii) reforzar las habilidades profesionales del personal involucrado en el programa de control de la esquistosomiasis. Los resultados de la simulación mostraron que esta estrategia podría acelerar el progreso del programa de control de la esquistosomiasis, pasando de la etapa de control a la etapa de eliminación.

Conclusiones: Los enfoques ecológicos implementados en las áreas endémicas de esquistosomiasis en la región de Eryuan son capaces de mejorar la eficacia conjunta de la protección ambiental y el control de la esquistosomiasis, proporcionando una nueva vía para la eliminación de la esquistosomiasis gracias a la aplicación de intervenciones precisas.

Translated from English version into Spanish by Eugenia Cagni, proofread by Kate Pattison, through

TRANSLATORS
WITHOUT BORDERS
