# Supplementary material for: Role of ecological approaches to eliminating schistosomiasis in Eryuan County evaluated by system modelling
Source: Infect Dis Poverty. 2018 Dec 20;7:129. doi: 10.1186/s40249-018-0511-7 (PMC6309097; doi:10.1186/s40249-018-0511-7)
Supplement: Supplementary file 2 — Diagram of main causal chains. (DOCX 43 kb) [file 40249_2018_511_MOESM2_ESM.docx]

**Additional file 2.**

**The diagram of the main causal chains of the System Modeling are as follows：**

Case detection

Health education

Livestock infection risk

The crowd infection risk

Area of snail

Disposal rate

Contact factor of contaminated water

Environment of contaminated water

The input in science and technology

Factor of science and technology

Schistosomiasis increment

Disposal rate

Contact factor of contaminated water

Environment of contaminated water

Input factor of science and technology

of contaminated water

Reduce the contact

Controlling the source of infection

Improve the ecological environment

Blocking the biological transmission chains

Schistosomiasis increment

Risk factors

Schistosomiasis index
