# Supplementary material for: Role of ecological approaches to eliminating schistosomiasis in Eryuan County evaluated by system modelling
Source: Infect Dis Poverty. 2018 Dec 20;7:129. doi: 10.1186/s40249-018-0511-7 (PMC6309097; doi:10.1186/s40249-018-0511-7)
Supplement: Supplementary file 3 — List of equations for statistic and function analyses in model development. (DOCX 24 kb) [file 40249_2018_511_MOESM3_ESM.docx]

**Additional file 3.**

1. **Model structure**

The basic structure of model is based on analyze the investment and benefit of schistosomiasis elimination measures. Choose the investment for state variables, including health education, health project, livestock project, ecological management in schistosomiasis elimination programme. By using Vensim table function of linear interpolation method to convert the disease transmission indicators into risk factors, including contact contaminated water, the human infection, livestock infection, etc.

Referring to the previous research results on weighting for factor indices in the evaluation, the previous transmission factors were translated into the index of schistosomiasis transmission which integrated into formation model (Figure 4). Finally, the simulation was undertaken by setting the growth rate of state variables in the model. The detail performance of modeling is described as follows.

**1.1 State variables**

The traditional control mode is to integrate efforts carried out by multi-sectors, including the health department, the department of agriculture and education department, then the water conservancy, forestry and other departments, in addition to the efforts on comprehensive treatment. After entered the stage of schistosomiasis transmission interruption, projects on health, livestock and health education are sustained by the government, but Eryuan County is launching a multi-sectoral integrated ecological management projects. So we choose the health, livestock, health eduction, and ecological management as the state variables for this study, use the project investment with unit of RMB in 2009 as initial data.

**1.2 Causal chain and auxiliary variables**

State variables according to the relevant causality are decomposed into control intervention of auxiliary variables, the main causal chain and auxiliary variables are as follows:

Health project - Check and cure disease - Risk of population infection - Infection risk factor

Health project - Snail searching and eradication - Area of snail - Infection risk factor

Health project - Scientific research - Scientific research input factor - Schistosomiasis incremental factor

Health project - Surveillance and reporting of cases - Schistosomiasis incremental factor

Livestock project - Livestock checking and treatment - Risk of livestock infection - Infection risk factor

Health education - Contagious water contact factor - Schistosomiasis incremental factor

Ecological management - Control the source of infection / Blocking transmission / Reduction of contact / Environmental improvement - Ecological factor

**1.3 Incremental and fixed parameters**

In order to realize the dynamic simulation of the model, the extension analysis timeline is needed to set the increment of state variable and rate of growth. Referring to the Eryuan county’s investment from 2009 to 2017, increment rate was set with its unit as year as the following formula:

Health/Livestock/Environment/Health education investment = INTEG (The input×Annual growth rate, The initial value)

In addition to the four incremental and growth rate, modeling was performed with also using some environment parameters as inputs including livestock checking and treatment proportion, risk monitoring, investment rate, scientific research proportion and so on.

**1.4 Risk factors and index**

Converting relevant variables to risk factor is performed by the table function of Vensim. Table function is an important feature of system dynamics, in order to establish the nonlinear relationship between two variables, especially soft variables, neat the relationship between the variables, draw a diagram, use means of linear interpolation, the value is a dimensionless quantity, and its expression is as follows:

Variable Y = X factor table variables (X)

X factor table = [(XLOW YLOW) - (XHIGH YHIGH)] (X1, Y1) (X2, Y2)...(Ym, Xn)

Independent variable values for XLOW (minimum) to XHIGH (maximum) with such as space between XINCR X1, X2,...Xm, m =（XHIGH-XLOW）/XINCR + 1，and

The numerical of TY is given with T equation: TY = E1 / E2 /..when X0∈（XLOW，XHIGH），but X0≠Xi（i=1,2,…,m）

The variable values given by the linear interpolation method. When the value of X is beyond the scope [XLOW XHIGH], the dependent variable values corresponding to the endpoint.

Through table function of Vensim, the variables transform was done from control interventions to the risk factors, such as risk of population infection, risk of livestock infection, contagious water contact and area of snail factor, as well as the scientific research input factor and regulate disposal rate. Then schistosomiasis transmission index is generated by the above factors according to the combined weight as description of Liu[25] and Xu[26] that all those combined weights were established by Delphi method in the schistosomiasis elimination programme. Those factors and relevant weights were listed in the Attached file 2 with the modeling development statistical data and function analysis of the equation. Those risk factors including the risk of population infection, risk of livestock infection, area of snail combination is for infection risk factor (positive feedback) were then combined with contaminated water (positive feedback), scientific research input factor (negative feedback) and regulate disposal rate (negative feedback) factor into schistosomiasis incremental factor (comprehensive feedback). Finally, above factors were combined with ecological factor (positive feedback) to form the schistosomiasis transmission index.

**2. The lists of the equations for the statistic and function analysis in the development of modeling are as follows:**

(01) FINAL TIME = 2030

Units: Year

The final time for the simulation.

(02) INITIAL TIME = 2007

Units: Year

The initial time for the simulation.

(03) SAVEPER = TIME STEP

Units: Year [0,?]

The frequency with which output is stored.

(04) TIME STEP = 1

Units: Year [0,?]

The time step for the simulation.

(05) Infection risk of the crowd= WITH LOOKUP(Check and cure the disease )

([(0,0)-(100,200)],(34,122),(36,193),(39,64),(42,148),(45,29),(48,21),(51,10),(100,0) ))

Units: Dmnl

(06) Health education= INTEG (increment of health education, 4.52)

Units: **undefined**

(07) Increment of health education = The growth rate of health education * health education

Units: Ten thousand yuan

(08) The growth rate of health education =0.4

Units: Dmnl

(09) Reduce the contact= the input in Ecological management *0.3

Units: Ten thousand yuan

(10) The input in Health lake= INTEG (increment of the input in Health lake,340)

Units: Ten thousand yuan

(11) Increment of the input in Health lake = the input in Health lake* growth rate of input

Units: **undefined**

(12) Growth rate of input =0.073

Units: **undefined**

(13) infection risk of Livestock = WITH LOOKUP (Check and cure for Livestock,([(0,0)-(100,200)],(27.78,5),(30.84,44),(31.94,98),(31.98,21),(32.76,38),(33,20) ))

Units: a

(14) Check and cure for Livestock = check proportion *the input in Livestock lake

Units: Ten thousand yuan

(15) The input in Livestock lake = INTEG (increment of the input in Livestock lake ,31.5)

Units: Ten thousand yuan

(16) Increment of the input in Livestock lake =( the input in Livestock lake + Health lake)*0.01

Units: Ten thousand yuan

(17) Disposal rate=INTEGER(Case detection *0.01)

Units: Dmnl

(18) Controlling the source of infection = the input in Ecological management *0.2

Units: Ten thousand yuan

(19) Improve the ecological environment = the input in Ecological management *0.2

Units: Ten thousand yuan

(20) Check proportion =0.6

Units: Dmnl

(21) Check and cure the disease = the input in Health lake *0.1

Units: **undefined**

(22) Snail control = the input in Health lake *0.2

Units: **undefined**

(23) The increase of Ecological management =the input in Ecological management*the growth rate of Ecological management

Units: Ten thousand yuan

(24) The input in Ecological management = INTEG ( the increase of Ecological management,2000)

Units: Ten thousand yuan

(25) The growth rate of Ecological management=0.2

Units: Dmnl

(26) contact factor of contaminated water = WITH LOOKUP (Health education,([(3,0)-(50,20),(3.58,14.64),(4.52,17.36),(6.72,11.3),(8.63,11.3),(8.75,10.33),(9.3,6.55)],(3.58,14.64),(4.52,17.36),(6.72,11.3),(8.63,11.3),(8.75,10.33),(9.3,6.55) ))

Units: Dmnl

(27) Environment of contaminated water =Livestock infection risk+The crowd infection risk + area of snail

Units: **undefined**

(28) Case detection = capacity building of schistosomiasis prevention /0.01+ Risk monitoring

Units: a

(29) Input factor of science and technology=INTEGER(The input in science and technology /10)

Units: Dmnl

(30) The percentage of science and technology =0.05

Units: Dmnl

(31) Input factor of science and technology =input in healthy lake* the percentage of science and technology

Units: Ten thousand yuan

(32) Schistosomiasis increment =environment of contaminated water- disposal rate - actor of science and technology+ contact factor of contaminated water

Units: Dmnl

(33) Schistosomiasis infection index = INTEG (((Schistosomiasis increment + risk factors),100)

Units: Dmnl

(34) Schistosomiasis index=(Schistosomiasis increment+ risk factors)/2

Units: **undefined**

(35) Proportion of Lake investment =0.05

Units: **undefined**

(36) Capacity building of schistosomiasis prevention = input in healthy lake * proportion of Lake investment

Units: Ten thousand yuan

(37) The area of snail = WITH LOOKUP (snail control,([(0,0)-(200,600)],(68,460),(72,395),(78,256),(84,149),(90,100) ))

Units: a

(38) Blocking the biological transmission chains

= the input in Ecological management *0.3

Units: Ten thousand yuan

(39) Risk factors = WITH LOOKUP (Reduce the contact+ Controlling the source of infection

+Improve the ecological environment +blocking the biological transmission chains,([(0,0)-(50000,400)],(2198,293),(2924,303),(6421,113),(7000,207),(8274,58),(9500,30),(9600,34),(40000,0) ))

Units: Dmnl

(40) Risk monitoring =1000

Units: a
