# Supplementary material for: Role of ecological approaches to eliminating schistosomiasis in Eryuan County evaluated by system modelling
Source: Infect Dis Poverty. 2018 Dec 20;7:129. doi: 10.1186/s40249-018-0511-7 (PMC6309097; doi:10.1186/s40249-018-0511-7)
Supplement: Supplementary file 4 — Statistically significant results obtained with SPSS. (DOCX 21 kb) [file 40249_2018_511_MOESM4_ESM.docx]

**Additional file 4.**

**Statistic results in significance test by using SPSS showing in 3 tables as follows:**

| **Table S1. Model Summary** | | | | | | | | | |
| --- | --- | --- | --- | --- | --- | --- | --- | --- | --- |
| Model | R | R Square | Adjusted R Square | Std. Error of the Estimate | Change Statistics | | | | |
|  |  |  |  |  | R Square Change | F Change | df1 | df2 | Sig. F Change |
| 1 | .971^a^ | .943 | .932 | 31.651 | .943 | 82.754 | 1 | 5 | .000 |
| a. Predictors: (Constant), Simulation value | | | | | | | | | |

| **Table S2. ANOVA^b^** | | | | | | |
| --- | --- | --- | --- | --- | --- | --- |
| Model | | Sum of Squares | df | Mean Square | F | Sig. |
| 1 | Regression | 82900.025 | 1 | 82900.025 | 82.754 | .000^a^ |
|  | Residual | 5008.832 | 5 | 1001.766 |  |  |
|  | Total | 87908.857 | 6 |  |  |  |
| a. Predictors: (Constant), Simulation value | | | | | | |
| b. Dependent Variable: true value | | | | | | |

| **Table S3. Coefficients^a^** | | | | | | |
| --- | --- | --- | --- | --- | --- | --- |
| Model | | Unstandardized Coefficients | | Standardized Coefficients | t | Sig. |
|  |  | B | Std. Error | Beta |  |  |
| 1 | (Constant) | -86.947 | 27.159 |  | -3.201 | .024 |
|  | Simulation value | 1.323 | .145 | .971 | 9.097 | .000 |
| a. Dependent Variable: true value | | | | | | |
